# Supplementary material for: Dose-Dependent Genetic Resistance to Azole Fungicides Found in the Apple Scab Pathogen
Source: J Fungi (Basel). 2023 Nov 24;9(12):1136. doi: 10.3390/jof9121136 (PMC10744243; doi:10.3390/jof9121136)
Supplement: Supplementary file 1 [file jof-09-01136-s001.zip › Tables S2-4.pdf]

Table S2. Lysis buffer: Constituents of buffers used to prepare lysis buffer for DNA extraction. Fresh lysis buffer was prepared as required prior to DNA extractions by combining stock preparations of BuffersA-E in the ratio 5:5:2:1:1 and adding 10 μL (10 kU) RNase A per 14 ml.

	**Constituents**			
**Buffer A**	0.35 M sorbitol	0.1 M TrisHCl	5 mM EDTA, pH 8	
**Buffer B**	0.2 M Tris-HCl	50 mM EDTA, pH 8	2 M NaCl	2% CTAB
**Buffer C**	5% Sarkosyl N-lauroylsarcosine sodium salt			
**Buffer D**	PVP40 10%			
**Buffer E**	PVP10 10%			

Table S3. Interior annotations for Linkage group seven Quantitative Trait Loci (QTLs): Gene ID, Genbank accession ID and any Gene Ontology (GO) terms associated with the predicted protein (InterPro search, default settings) are given for each prediction, starred (*) gene IDs contain non-synonymous single nucleotide polymorphisms.

**Gene ID:**	**GenBank Accession:**	**Gene Ontology terms:**
Vi05172_g2743*	RDI87257.1	None
Vi05172_g2744	RDI87264.1	None
Vi05172_g2745	RDI87263.1	None
Vi05172_g2746*	RDI87262.1	None
Vi05172_g2747	RDI87261.1	polysaccharide catabolic process (GO:0000272); hydrolase activity, hydrolyzing O-glycosyl compounds (GO:0004553); cellulase activity (GO:0008810)
Vi05172_g2748	RDI87266.1	None
Vi05172_g2749	RDI87265.1	None
Vi05172_g2750	RDI87084.1	nucleic acid binding (GO:0003676); ATP binding (GO:0005524)
Vi05172_g2751	RDI87085.1	None
Vi05172_g2752	RDI87086.1	protein import into peroxisome matrix (GO:0016558); protein binding (GO:0005515); zinc ion binding (GO:0008270); peroxisomal membrane (GO:0005778)
Vi05172_g2753	RDI87087.1	None
Vi05172_g2754	RDI87080.1	None
Vi05172_g2755*	RDI87081.1	sulfate transport (GO:0008272); transmembrane transport (GO:0055085); secondary active sulfate transmembrane transporter activity (GO:0008271); membrane (GO:0016020)
Vi05172_g2756	RDI87082.1	None
Vi05172_g2757	RDI87083.1	steroid biosynthetic process (GO:0006694); 3-beta-hydroxy-delta5-steroid dehydrogenase activity (GO:0003854); oxidoreductase activity, acting on the CH-OH group of donors, NAD or NADP as acceptor (GO:0016616)
Vi05172_g2758*	RDI87078.1	ATP binding (GO:0005524); ATP hydrolysis activity (GO:0016887)
Vi05172_g2759	RDI87079.1	N-acetyltransferase activity (GO:0008080); acyltransferase activity, transferring groups other than amino-acyl groups (GO:0016747)
Vi05172_g2760*	RDI87242.1	ATP binding (GO:0005524); ATP-dependent chromatin remodeler activity (GO:0140658)
Vi05172_g2762*	RDI87244.1	None
Vi05172_g2763	RDI87243.1	None
Vi05172_g2764	RDI87238.1	gamma-glutamylcyclotransferase activity (GO:0003839)
Vi05172_g2765	RDI87237.1	None
Vi05172_g2766	RDI87240.1	None
Vi05172_g2767*	RDI87239.1	None
Vi05172_g2768	RDI87246.1	None
Vi05172_g2769	RDI87245.1	transmembrane transport (GO:0055085); transmembrane transporter activity (GO:0022857)
Vi05172_g2770	RDI87064.1	None
Vi05172_g2771	RDI87065.1	None

Vi05172_g2772	RDI87062.1	mitochondrial pyruvate transmembrane transport (GO:0006850); mitochondrial inner membrane (GO:0005743)
Vi05172_g2773	RDI87063.1	gluconeogenesis (GO:0006094); pyruvate metabolic process (GO:0006090); ATP binding (GO:0005524); pyruvate carboxylase activity (GO:0004736); catalytic activity (GO:0003824); metal ion binding (GO:0046872)
Vi05172_g2774	RDI87060.1	None
Vi05172_g2775	RDI87061.1	vesicle-mediated transport (GO:0016192); intracellular protein transport (GO:0006886); structural molecule activity (GO:0005198); protein binding (GO:0005515); clathrin light chain binding (GO:0032051); clathrin coat of trans-Golgi network vesicle (GO:0030130); clathrin coat of coated pit (GO:0030132); clathrin complex (GO:0071439)
Vi05172_g2776*	RDI87058.1	translation initiation factor activity (GO:0003743); cytoplasm (GO:0005737); eukaryotic translation initiation factor 3 complex (GO:0005852)
Vi05172_g2777	RDI87059.1	translation (GO:0006412); structural constituent of ribosome (GO:0003735); ribosome (GO:0005840)
Vi05172_g2779*	RDI87067.1	None
Vi05172_g2780	RDI87348.1	glycolytic process (GO:0006096); magnesium ion binding (GO:0000287); phosphopyruvate hydratase activity (GO:0004634); phosphopyruvate hydratase complex (GO:0000015)
Vi05172_g2782	RDI87346.1	None
Vi05172_g2783*	RDI87345.1	GTP binding (GO:0005525); GTPase activity (GO:0003924); tRNA binding (GO:0000049)
Vi05172_g2785	RDI87344.1	amino acid transport (GO:0006865); transmembrane transport (GO:0055085); membrane (GO:0016020)
Vi05172_g2786	RDI87343.1	None
Vi05172_g2787	RDI87342.1	transmembrane transport (GO:0055085); transmembrane transporter activity (GO:0022857); membrane (GO:0016020)
Vi05172_g2788	RDI87341.1	oxidoreductase activity (GO:0016491)
Vi05172_g2789	RDI87340.1	None
Vi05172_g2790*	RDI87163.1	zinc ion binding (GO:0008270)
Vi05172_g2791	RDI87361.1	None
Vi05172_g2792	RDI87164.1	RNA processing (GO:0006396); protein binding (GO:0005515)
Vi05172_g2793	RDI87165.1	None
Vi05172_g2794	RDI87166.1	None
Vi05172_g2795	RDI87167.1	None
Vi05172_g2796	RDI87168.1	calcium ion transport (GO:0006816); nucleotide binding (GO:0000166); P-type calcium transporter activity (GO:0005388); ATP binding (GO:0005524); ATP hydrolysis activity (GO:0016887); transporter activity (GO:0005215); membrane (GO:0016020)
Vi05172_g2797	RDI87169.1	None
Vi05172_g2798	RDI87161.1	None
Vi05172_g2799	RDI87162.1	None
Vi05172_g2800*	RDI87170.1	flavin adenine dinucleotide binding (GO:0050660); FAD binding (GO:0071949); oxidoreductase activity (GO:0016491)
Vi05172_g2802	RDI87172.1	None

Vi05172_g2804*	RDI87174.1	regulation of DNA-templated transcription (GO:0006355); DNA-templated transcription (GO:0006351); zinc ion binding (GO:0008270); DNA-binding transcription factor activity, RNA polymerase II-specific (GO:0000981); DNA binding (GO:0003677)
Vi05172_g2806*	RDI87176.1	None
Vi05172_g2807*	RDI87177.1	protein binding (GO:0005515)
Vi05172_g2808	RDI87178.1	None
Vi05172_g2810*	RDI87309.1	tRNA wobble uridine modification (GO:0002098); elongator holoenzyme complex (GO:0033588)
Vi05172_g2812*	RDI87354.1	chloride transport (GO:0006821); transmembrane transport (GO:0055085); voltage-gated chloride channel activity (GO:0005247); membrane (GO:0016020)
Vi05172_g2814	RDI87352.1	trehalose biosynthetic process (GO:0005992); catalytic activity (GO:0003824)
Vi05172_g2815	RDI87351.1	protein phosphorylation (GO:0006468); mRNA processing (GO:0006397); endoplasmic reticulum unfolded protein response (GO:0030968); ATP binding (GO:0005524); protein kinase activity (GO:0004672); RNA nuclease activity (GO:0004540); RNA endonuclease activity (GO:0004521); protein serine/threonine kinase activity (GO:0004674); protein binding (GO:0005515)
Vi05172_g2816*	RDI87350.1	None
Vi05172_g2817	RDI87349.1	None
Vi05172_g2818*	RDI87357.1	transmembrane transport (GO:0055085); ATP binding (GO:0005524); ABC-type transporter activity (GO:0140359); ATP hydrolysis activity (GO:0016887); membrane (GO:0016020)
Vi05172_g2819	RDI87356.1	None
Vi05172_g2821	RDI87156.1	ubiquinone biosynthetic process (GO:0006744); cellular respiration (GO:0045333); ubiquinone binding (GO:0048039)
Vi05172_g2822	RDI87153.1	None
Vi05172_g2823*	RDI87154.1	S-adenosylmethionine-dependent methyltransferase activity (GO:0008757)

Table S4. Interior annotations for Linkage group one Quantitative Trait Loci (QTLs): Gene ID, Genbank accession ID and any Gene Ontology (GO) terms associated with the predicted protein (InterPro search, default settings) are given for each prediction, starred (*) gene IDs contain non-synonymous single nucleotide polymorphisms.

Gene ID:	GenBank Accession:	Gene Ontology terms:
Vi05172_g3975	RDI85793.1	None
Vi05172_g3976*	RDI85792.1	transmembrane transport (GO:0055085); transmembrane transporter activity (GO:0022857); electron transfer activity (GO:0009055); oxidoreductase activity, acting on the aldehyde or oxo group of donors, iron-sulfur protein as acceptor (GO:0016625); iron-sulfur cluster binding (GO:0051536); oxidoreductase activity (GO:0016491)
Vi05172_g3977	RDI85791.1	None
Vi05172_g3978	RDI85800.1	protein phosphorylation (GO:0006468); autophagy (GO:0006914); protein kinase activity (GO:0004672); ATP binding (GO:0005524); protein serine/threonine kinase activity (GO:0004674)
Vi05172_g3979	RDI85799.1	None
Vi05172_g3980*	RDI85949.1	None
Vi05172_g3981*	RDI85950.1	oxidoreductase activity (GO:0016491)
Vi05172_g3982*	RDI85947.1	carbohydrate metabolic process (GO:0005975); hydrolase activity, hydrolyzing O-glycosyl compounds (GO:0004553); alpha-galactosidase activity (GO:0004557)
Vi05172_g3983*	RDI85948.1	None
Vi05172_g3984*	RDI85945.1	transmembrane transport (GO:0055085); transmembrane transporter activity (GO:0022857)
Vi05172_g3985*	RDI85946.1	chloride channel activity (GO:0005254)
Vi05172_g3986*	RDI85943.1	telomere capping (GO:0016233); single-stranded telomeric DNA binding (GO:0043047); CST complex (GO:1990879)
Vi05172_g3989*	RDI85942.1	None
Vi05172_g3990	RDI85809.1	None
Vi05172_g3991	RDI85808.1	None
Vi05172_g3992	RDI85811.1	None
Vi05172_g3993	RDI85810.1	None
Vi05172_g3994*	RDI85805.1	oxidoreductase activity, acting on single donors with incorporation of molecular oxygen, incorporation of two atoms of oxygen (GO:0016702)
Vi05172_g3995	RDI85804.1	None
Vi05172_g3998	RDI85803.1	transmembrane transport (GO:0055085); transmembrane transporter activity (GO:0022857); membrane (GO:0016020)
Vi05172_g3999	RDI85802.1	None
Vi05172_g4001	RDI85995.1	carbohydrate metabolic process (GO:0005975); carbohydrate binding (GO:0030246); catalytic activity (GO:0003824); isomerase activity (GO:0016853)
Vi05172_g4002*	RDI85992.1	None
Vi05172_g4003	RDI85993.1	None
Vi05172_g4004	RDI85990.1	None
Vi05172_g4005	RDI85991.1	transmembrane transport (GO:0055085)

Vi05172_g4006*	RDI85988.1	protein phosphorylation (GO:0006468); protein kinase activity (GO:0004672); ATP binding (GO:0005524)
Vi05172_g4007*	RDI85989.1	None
Vi05172_g4008*	RDI85986.1	ubiquitin-dependent protein catabolic process (GO:0006511); ubiquitin-protein transferase activity (GO:0004842); ubiquitin protein ligase activity (GO:0061630)
Vi05172_g4009*	RDI85987.1	organic substance metabolic process (GO:0071704); hydrolase activity, hydrolyzing O-glycosyl compounds (GO:0004553)
Vi05172_g4010	RDI85826.1	None
Vi05172_g4011	RDI85825.1	None
Vi05172_g4012	RDI85828.1	DNA repair (GO:0006281); metal ion binding (GO:0046872); DNA binding (GO:0003677)
Vi05172_g4013*	RDI85827.1	None
Vi05172_g4014*	RDI85823.1	carbohydrate metabolic process (GO:0005975)
Vi05172_g4015*	RDI85822.1	RNA helicase activity (GO:0003724); ATP binding (GO:0005524); nucleic acid binding (GO:0003676)
Vi05172_g4016*	RDI85801.1	None
Vi05172_g4017	RDI85824.1	carboxylic acid metabolic process (GO:0019752); catalytic activity (GO:0003824); isocitrate lyase activity (GO:0004451)
Vi05172_g4018	RDI85830.1	None
